# A173 ELUCIDATING THE EFFECTS OF NOD2-MEDIATED SIGNALLING ON INTESTINAL RESIDENT-MEMORY T-CELL FORMATION AND FUNCTION

**DOI:** 10.1093/jcag/gwac036.173

**Published:** 2023-03-07

**Authors:** B Tsankov, C Carr, A Luchak, N Nathan, S Girardin, D Philpott

**Affiliations:** 1 Immunology; 2 Laboratory Medicine and Pathobiology, Medical Sciences Building, University of Toronto, Toronto, Canada

## Abstract

**Background:**

Aberrant resident memory T-cell (TRM) responses have been associated with increased intestinal inflammation and Crohn’s disease (CD) pathology in humans. Intestinal TRM cells are not only important for maintaining the integrity of the intestinal epithelial barrier, but also for the rapid clearance of pathogens in the intestine during infection. Understanding the signals received by the intestinal immune system to generate TRM responses is paramount to elucidating treatments for CD. Genetic mutations in *NOD2* are associated with the highest risk of CD development. As a host intracellular sensor of bacterial peptidoglycan, NOD2 is critical for initiating both innate and adaptive immune responses. Furthermore, work from our lab as well as those of our collaborators suggest that *NOD2* deficiency reduces systemic memory B and T-cell responses. However, the role of NOD2 in establishing memory T-cell responses in the intestine remains unclear. This work will therefore establish the role of NOD2 signaling in initiating and maintaining optimal TRM responses to achieve intestinal homeostasis and resilience to intestinal inflammation.

**Purpose:**

It is the main objective of this project to determine whether NOD2-mediated signalling affects:

1. Antigen-specific T-cell priming *in vivo*

2. *Bona fide* intestinal T_RM_ generation

3. *Bona fide* intestinal T_RM_ function

**Method:**

To address the effects of NOD2-signalling on intestinal T-cell priming in vivo, wildtype (WT) mice were adoptively transferred 50,000 naïve LCMV-specific (SMARTA) CD4^+^ T-cells. Mice were subsequently infected with LCMV-Armstrong in the presence or absence of the NOD2 agonist; MDP. 5-days following infection, the numbers and percentage of LCMV-specific T-cells in the mesenteric lymph nodes and spleen were examined. To examine the effects of NOD2 on intestinal T_RM_ generation, littermate WT and NOD2 KO mice were infected with LCMV-Armstrong. Thirty-six-days following infection, the percentage and number of LCMV-specific CD4^+^ T-cells were profiled in the small and large intestinal lamina-propria by means of gp_66-77_ class-II MHC-tetramer staining. In another set of experiments, littermate WT and NOD2 KO mice were re-infected with LCMV-C13 30-days following LCMV-Armstrong immunization, and the interferon-response in the small intestine was profiled by quantitative PCR to assess the effect of NOD2-deficiency on antigen recall responses.

**Result(s):**

NOD2-stimulation by means of MDP injection increased the percentage and number of adoptively transferred SMARTA CD4^+^ T-cells in the mesenteric lymph nodes upon LCMV infection. Furthermore, NOD2-deficiency did not alter intestinal LCMV-specific CD4^+^ T_RM_ seeding in the small and large intestinal lamina propria 36 days after infection. However, *in vivo* antigen recall experiments showed a decreased intestinal IFN response in NOD2 KO mice.

**Conclusion(s):**

Our findings reveal a potential role of NOD2 in the intestinal CD4^+^ T-cell priming and subsequent Ag-specific memory response.

**Disclosure of Interest:**

None Declared

